# Annotations

**Published:** 1908-04-04

**Authors:** 


### Annotations.

### Flies, Dust, and Mosquitoes.

One of the most encouraging signs of the times from a hygienic point of view is the space devoted in the lay press to various points connected with the spread of disease. During the last few days readers of our daily press have been presented with a good many facts of this kind, particularly on those prolific agents of morbidity—flies, dust and mosquitoes. The press would not notice the various discussions on such subjects unless the public was genuinely interested therein. On flies as carriers of disease Dr. Hamer presented an exhaustive report to the London County Council, in which he concludes that though it is not yet possible to express a positive opinion as to disease, it is certain that contamination of food, irritation and annoyance, even loss of sleep, are greatly increased in the immediate proximity of refuse depôts and other trade premises likely to promote the breeding of flies. On dust we have had a discussion at a meeting of the Royal Sanitary Institute in connection with the question of road requirements. The dissemination of disease by dust was included in the debate, and it is highly satisfactory to know that engineers are taking the problem into their consideration. On mosquitoes popular attention has once more been focussed by abstracts of the evidence of Professor Osler before the Royal Commission of Vivisection on the subject of yellow fever, and the experiments at Havannah by which the part played by mosquitoes in disseminating it was discovered. Dangerous though the quack puffs and paragraphs of the less reputable press usually are, we have nothing but eulogy for the presentation to the people of up-to-date information on hygienic subjects, provided always that accuracy is not sacrificed to sensationalism.

### The Disposal of the Dead.

The history of cremation in England is curious and interesting. When this practice was first advocated in 1874 by Sir Henry Thompson it was supported by a little band of enthusiasts, while against it was ranged the bitterest and most determined opposition. It seemed, indeed, for some time as though the movement must inevitably be crushed by the sheer weight and number of the attacking forces. But that is over, and active hostility to cremation is a thing of the past. The process is now acknowledged to be simple, expeditious, hygienic, and reverent. Moreover, when contrasted with earth-burial of the same standard of decency, in districts accessible to crematoria, it is actually economical. In spite of all this, however, the progress of cremation is disappointingly slow. The old unreasoning loyalty to the slow corruption of the grave survives in the heart of the average Englishman. It is only among the wealthy and the intellectual that cremation is gaining ground. One might have supposed that, as soon as prejudice was swept away, fire would displace the worm; but it is now clear that, so far as the public is concerned, active opposition has merely yielded to passive acquiescence, and widespread practical support can only be secured by vigorous methods. Incineration must be made even cheaper than it is, and much more accessible; but, above all, its advantages must be published far and wide. It is not enough to have vindicated cremation in the eyes of the *élite*: their example will be fruitless so long as the average citizen looks upon this practice as a costly and rather fantastic luxury. Recognising that the time has come for greater publicity and renewed exertions, the Cremation Society of England has decided to lower its membership fees and otherwise to extend its operations. It is important that all medical men should treat this question seriously, because the weight of their opinion and influence will largely determine the future course of the movement. Should cremation displace earth-burial as the usual practice in this country, drastic reforms in the methods of death certification must necessarily follow: and this seems to us to be an additional argument in favour of cremation.

### Anæsthetics in America.

The subject of anæsthesia and its dangers seems to be attracting considerable attention on the other side of the Atlantic as well as in our own country. Dr. John B. Roberts, in the *Therapeutic Gazette*, gives a searching criticism of the usual anæsthetic procedure in American hospitals. In his opinion the United States are far behind Great Britain in safeguarding surgical patients from the perils of anæsthesia. He speaks of a "reckless disregard of the risks of anæsthetic inhalation" on the part of the medical staffs and lay managers of hospitals. The principal faults, according to Dr. Roberts, are six in number: The employment of inexperienced administrators; deficient teaching of anæsthetics; the induction of general anæsthesia when local anæsthesia or no anæsthesia at all is indicated; excessive dosage; insufficient care in watching symptoms of danger during the operation; and neglect in keeping close watch upon the patient after the operation has been completed. Dr. Roberts agrees with certain English authorities in demanding more thorough instruction in anæsthetic administration in medical schools, and increased facilities for men to equip themselves as "anæsthesia specialists." He suggests that resident anæsthetists should be attached to all American hospitals at a proper salary, and that in private operations the skilled anæsthetist should receive direct from the patient a fee equal to one-tenth of that charged by the surgeon for the operation itself. The present dearth of specially qualified and experienced anæsthetists exclusively engaged upon this branch of practice is in the author's opinion a grave defect in American surgery which should be remedied at once. He alleges that the number of unnecessary deaths from anæsthetics in that country is larger than is realised.

